# A statistical model for the identification of genes governing the incidence of cancer with age

**DOI:** 10.1186/1742-4682-5-7

**Published:** 2008-04-16

**Authors:** Kiranmoy Das, Rongling Wu

**Affiliations:** 1 Department of Statistics, University of Florida, Gainesville, FL 32611, USA; 2 UF Genetics Institute, University of Florida, Gainesville, FL 32611, USA; 3 Department of Operation Research and Financial Engineering, Princeton University, Princeton, NJ 08544, USA

## Abstract

The cancer incidence increases with age. This epidemiological pattern of cancer incidence can be attributed to molecular and cellular processes of individual subjects. Also, the incidence of cancer with ages can be controlled by genes. Here we present a dynamic statistical model for explaining the epidemiological pattern of cancer incidence based on individual genes that regulate cancer formation and progression. We incorporate the mathematical equations of age-specific cancer incidence into a framework for functional mapping aimed at identifying quantitative trait loci (QTLs) for dynamic changes of a complex trait. The mathematical parameters that specify differences in the curve of cancer incidence among QTL genotypes are estimated within the context of maximum likelihood. The model provides testable quantitative hypotheses about the initiation and duration of genetic expression for QTLs involved in cancer progression. Computer simulation was used to examine the statistical behavior of the model. The model can be used as a tool for explaining the epidemiological pattern of cancer incidence.

## Background

Age is thought to be the largest single risk factor for developing cancer [1,2]. A considerable body of data suggests that the incidence of cancer increases exponentially with age [3-7], although death from cancer may decline at very old age. This age-dependent rise in cancer incidence is characteristic of multicellular organisms that contain a large proportion of mitotic cells. For those organisms composed primarily of postmitotic cells, such as *Drosophila melanogaster *(flies) and *Caenorhabditis elegans *(worms), no cancer will develop. Elucidation of the causes of increasing cancer incidence with age in multicellular organisms can help to design a strategy for primary cancer prevention. The association between cancer and age can be explained by one or two of the physiological causes [8], i.e., a more prolonged exposure to carcinogens in older individuals [9] and an increasingly favorable environment for the induction of neoplasms in senescent cells [10]. These two possible causes lead older humans to accumulate effects of mutational load, increased epigenetic gene silencing, telomere dysfunction, and altered stromal milieu [2].

As a complex biological phenomena, susceptibility to cancer and its age-dependent increase is thought to include mixed genetic and environmental components [11-13]. The use of candidate gene approaches or association studies has led to the identification of specific genetic variants for cancer risk and their interactions with other genes and with environment, such as lifestyle. A more powerful method for cancer gene identification is to scan the complete genome for polymorphisms that confer increased risk [11]. Genome-wide identification of cancer genes has been conducted in laboratory mice by mapping individual quantitative trait loci (QTLs) for tumor susceptibility or resistance [12-14]. As a model system for studying human cancer, mice have been useful for elucidating the genetic architecture of cancer through the control of environmental exposure leading to tumorigenesis, which cannot be done with human populations [11]. A recent success in constructing a haplotype map of the human genome with single nucleotide polymorphisms (SNPs) [15] will make it possible to conduct a similar genome-wide search at the DNA sequence level in humans, as long as a statistical method that can detect the association between cancer and genes is available.

Unlike a static trait, age-related progressive changes in cancer incidence are a dynamic process. For this reason, traditional methods for QTL mapping of static traits will not be feasible, at least not be efficient, because the temporal pattern of cancer incidence is not considered. Recently, Wu and colleagues have developed a series of statistical models for mapping dynamic traits in which mathematical functions that specify biological processes are integrated into a QTL mapping framework (reviewed in [16]). The basic principle of these models, called functional mapping, is to characterize the genetic effects of QTLs on the formation and process of a biological trait by estimating and testing genotype-specific mathematical parameters for dynamic processes. Functional mapping is now used to map QTLs for growth curves in experimental crosses through linkage analysis [17-20] and for HIV dynamics and circadian rhythms in natural populations though linkage disequilibrium analysis [21-23].

In this article, we attempt to extend the idea of functional mapping to detect QTLs that predispose organisms to an age-related rise in cancer incidence. Frank [4] proposed a mathematical model for the age-specific incidence of cancer based on the molecular processes that lead to uncontrolled cellular proliferation. This model is defined by two key parameters, carrying capacity (*K*) and intrinsic growth rate (*r*). Thus, by estimating genotype-specific differences in these two parameters, the genetic effect of a QTL on age-related increase in cancer incidence can be estimated and tested. The new model will be designed for mouse systems, in which cancer cells can be counted in lifetime. Also, by controlling the environment of mouse models, the new model is able to understand how a QTL interacts with environmental carcinogens to produce cancer. For experimental crosses derived from inbred strains of mice, linkage mapping based on the estimation of the recombination fractions between different loci can serve a genome-wide search for cancer QTLs [24,25]. For outbred or wild mice that containing multiple genotypes, cancer QTL identification can be based on linkage disequilibrium analysis [26]. The new model for cancer incidence will be constructed with a random sample drawn from an experimental or natural population in which genetic markers are associated with the underlying QTL in terms of linkage disequilibrium. The new model provides a number of biologically meaningful hypothesis tests about the genetic and developmental control mechanisms underlying cancer risk. Computer simulations were performed to investigate the statistical behavior of the new model and validate its utilization.

## Model

### Logistic Model

It is well known that the incidence of cancer increases progressively with age [3]. This epidemiological pattern of cancer incidence is rooted in mutational processes. By assuming that cancer arises through the sequential accumulation of mutations within cell lineages [27], Frank [4,28] provided a general mathematical (logistic) equation for describing age-specific clonal expansion resulting from a mutation. Starting with a single cell, the number of clonal cells due to accumulative mutations after a time period *t *is expressed as

$$y(t)=\frac{Ke^{rt}}{K+e^{rt}-1},$$

where *K *is the carrying capacity and *r *is the intrinsic rate of increase of the clone. If a QTL affects age-dependent clonal expansion, there will be different carrying capacities and different rates of increase among different QTL genotypes.

### Mapping Population

Suppose there are two groups of mice randomly drawn from an experimental or natural population at Hardy-Weinberg equilibrium. These two groups are reared in two different controlled environments, such as case (the mice exposed to a carcinogen) and control (with no such exposure). Let *n*_*k *_be the size of group *k *(*k *= 1, 2). For both groups, molecular markers such as single nucleotide polymorphisms (SNPs) are genotyped throughout the genome. For each sampled mouse in each group, the number of cells in the clone due to accumulated mutations is counted at a series of equally-spaced ages, (1, 2, ..., *T*), in lifetime.

Assume that a QTL with alleles *A *and *a *affects the clonal expansion of cells. This QTL is associated with a marker with alleles *M *and *m*. The linkage disequilibrium between the QTL and marker is denoted as *D*. Let *p*, 1 - *p *and *q*, 1 - *q *be the frequencies of marker alleles *M*, *m *and QTL alleles *A*, *a*, respectively, in the population. The QTL and marker generate four haplotypes, *MA*, *Ma*, *mA *and *ma*. The frequencies of these haplotypes are expressed, respectively, as

*p*_11 _= *pq *+ *D*,

*p*_10 _= *p*(1 - *q*) - *D*,

*p*_01 _= (1 - *p*)*q *- *D*,

*p*_00 _= (1 - *p*)(1 - *q*) + *D*.

These haplotype frequencies are used to derive the joint genotype frequencies of the marker and QTL, expressed as

$$\begin{array}{llll} & AA & Aa & aa\\ MM & p_{11}^{2} & 2p_{11}p_{10} & p_{01}^{2}\\ Mm & 2p_{11}p_{01} & 2p_{11}p_{00}+2p_{10}p_{01} & 2p_{01}p_{00}\\ mm & p_{01}^{2} & 2p_{01}p_{00} & p_{00}^{2} \end{array}$$

from which we can derive the conditional probabilities of a QTL genotype, *j *(*j *= 0 for *aa*, 1 for *Aa *and 2 for *AA*), given a marker genotype of subject *i*, symbolized as *ω*_*j*|*i*_. Conditional probability *ω*_*j*|*i *_is a function of **Ω **= (*p*, *q*, *D*).

### Likelihood

For subject *i*, the number of clonal cells at age *t *(*t *= 1, 2, ..., *T*) under environment *k *can be expressed in terms of the underlying QTL as

*y*_*ik*_(*t*) = *ξ*_*i*0_*g*_0*k*_(*t*) + *ξ *_*i*1*g*1*k*_(*t*) + *ξ*_*i*2*g*2*k*_(*t*) + *e*_*ik*_(*t*),

where *ξ*_*ij *_is an indicator variable for a possible QTL genotype of individual *i*, defined as 1 if a particular QTL genotype *j *is indicated and 0 otherwise; *g*_*jk*_(*t*) is the genotypic value of QTL genotype *j *for clonal number at age *t*, which can be fit by Frank's [4] logistic model, i.e.,

$$g_{jk}(t)=\frac{K_{jk}e^{r_{jk}t}}{K_{jk}+e^{r_{jk}t}-1}$$

specified by a set of parameters $\Theta ={\left\{\Theta_{jk}\right\}}_{j=0,k=1}^{2,2}={\left\{K_{jk},r_{jk}\right\}}_{j=0,k=1}^{2,2}$ and *e*_*ik*_(*t*) is the residual effect for subject *i*, distributed as MVN(0, **Σ_*i*_**). We assume that matrix **Σ_*i *_**is composed of the two covariance matrices each under a different environment (*k*) since covariances between environments are thought not to exist. The covariance matrix under environment *k *is fit by a first-order autoregressive (AR(1)) model with variance $\sigma_{k}^{2}$ and correlation *ρ*_*k *_arrayed in ***Ψ ***= {***Ψ***_*k*_}.

The mixture model-based likelihood of samples with longitudinal measurements **y **and marker information **M **is formulated as

$$L(\Omega ,\Theta ,\Psi |\text{y},\text{M})=\prod_{k=1}^{2}\prod_{i=1}^{n_{k}}\left[\sum_{j=0}^{2}\omega_{j|i}f_{jk}({\text{y}}_{ik})\right]$$

where *f*_*jk*_(*y*_*ik*_) is a multivariate normal distribution for the number of clonal cells with mean vectors specified by **Θ**_*jk *_and covariance matrix specified by the AR(1) model with ***Ψ***_*k*_.

### Estimation and Algorithm

The likelihood (3) contains three types of parameters (**Ω**, **Θ, *Ψ ***), which can be estimated by the EM algorithm or simplex algorithm. Wang and Wu [21] derived a closed form for the EM algorithm to obtain the maximum likelihood estimates (MLEs) of the haplotype frequencies, and therefore the allele frequencies and linkage disequilibrium contained in **Ω**. Because age-dependent means and covariances are modeled by non-linear equations, it is difficult to derive the closed forms for these model parameters. Wang and Wu [21] have successfully used the simple algorithm to obtain the MLEs of parameters contained in **Θ **and ***Ψ***.

## Hypothesis Testing

One of the most significant advantages of functional mapping is that it can ask and address biologically meaningful questions by formulating a series of statistical hypothesis tests. Here, we describe the most important hypotheses as follows:

### Existence of a QTL

Testing whether a specific QTL is associated with the logistic function of the number of clonal cells is a first step toward understanding the genetic architecture of clonal expansion. The genetic control of the entire clonal expansion process can be tested by formulating the hypothesis:

*H*_0 _: *D *= 0 vs. *H*_1 _: *D ≠ *0.

The null hypothesis states that there is no QTL affecting the clonal expansion of the cells (the reduced model), whereas the alternative states that such a QTL does exist (the full model). The statistic for testing this hypothesis is the log-likelihood ratio (LR) of the reduced to the full model, i.e.,

$$LR_{1}=-2[\ln L(\tilde{\Omega },\tilde{\Theta },\tilde{\Psi })-\ln L(\tilde{\Omega },\tilde{\Theta },\tilde{\Psi })],$$

where the tildes and hats denote the MLEs of the unknown parameters under the *H*_0 _and *H*_1_, respectively. The LR is asymptotically *χ *^2^-distributed with one degree of freedom.

A similar test for the existence of a QTL can be performed on the basis of the hypotheses about genotypic-specific differences in curve parameters, i.e.,

$$\begin{array}{ll} H_{0}: & \Theta_{jk}\equiv (K,r),j=0,1,2;k=1,2\\ H_{1}: & \text{At least one of the equalities in }H_{0}\text{ does not hold}. \end{array}$$

We can compute the LR by calculating the parameter estimates under the null and alternative hypotheses above. However, in this case, it is difficult to determine the distribution of the LR because linkage disequilibrium is not identifiable under the null. An empirical approach to determine the critical threshold is based on permutation tests, as suggested by Churchill and Doerge [29].

Although the two hypotheses (4 and 6) can be used to test the existence of a QTL in association with a genotyped marker, they have a different focus. The null hypothesis of (4) proposes that a QTL may exist, but it is not associated with the marker. The null hypothesis of (6) states that no significant QTL exists, regardless of its association with the marker. Because of this difference, the critical value for the LR calculated under Hypothesis (4) can be determined from a *χ*^2^-distribution, whereas permutation tests are used to determine the critical value under Hypothesis (6) because the LR distribution is unknown.

### Pleiotropic Effect of the QTL

If a significant QTL is found to exist, the next test is for a pleiotropic effect of this QTL on clonal expansion under two different environments. The effects of this QTL expressed in environments 1 and 2 are tested by

$$\begin{array}{ll} H_{0}: & \Theta_{j1}\equiv (K_{1},r_{1}),j=0,1,2\\ H_{1}: & \text{At least one of the equalities in }H_{0}\text{ does not hold,} \end{array}$$

and

$$\begin{array}{ll} H_{0}: & \Theta_{j2}\equiv (K_{2},r_{2}),j=0,1,2\\ H_{1}: & \text{At least one of the equalities in }H_{0}\text{ does not hold}\text{.} \end{array}$$

If both the null hypotheses above are rejected, this means that the detected QTL exerts a pleiotropic effect on clonal expansion in the two environments considered. The thresholds for these tests can be determined from permutation tests separately for different environments.

### QTL by Environment Interaction

If the QTL shows a significant effect only in one environment, this means that a significant QTL by environment interaction exists. However, a pleiotropic QTL may also show significant QTL by environment interactions, depending on whether there is a difference in age-specific genetic effects between the two environments. This can be tested by formulating the following hypotheses:

$$\begin{array}{ll} H_{0}: & \Theta_{01}+\Theta_{21}=\Theta_{02}+\Theta_{22}\text{ and }2\Theta_{11}-(\Theta_{01}+\Theta_{21})=2\Theta_{12}-(\Theta_{02}+\Theta_{22})\\ H_{1}: & \text{At least one of the equalities in }H_{0}\text{ does not hold,} \end{array}$$

The critical value for the testing QTL by environment interactions can be based on simulation studies.

### Testing for Individual Parameters

Our hypotheses can also be based on individual parameters (*K *and *r*) that determine age-related changes for the numbers of clonal cells. We can test how a QTL affects each of these two parameter, and whether there is a significant QTL by environment interaction for each parameter. The critical values for these tests can be based on simulation studies.

## Computer Simulations

We perform simulation experiments to examine the statistical properties of the model proposed to detect QTLs responsible for clonal expansion. We assume an experimental or natural mouse population that is at Hardy-Weinberg equilibrium. A molecular marker with two alleles *M *and *m *is associated with a QTL with two alleles *A *and *a *that determines the clonal expansion of a cancer with age. The allele frequencies of marker allele *M *and QTL allele *Q *are assumed to be *p *= 0.5 and *q *= 0.6, respectively, and there is a positive value of linkage disequilibrium (*D *= 0.08) between the marker and the QTL. Using these allele frequencies and linkage disequilibrium, the distribution and frequencies of marker-QTL genotypes in the population can be simulated.

In order to study the genetic control of cancer incidence, we select a panel of mice randomly from the population and divide them into two groups, each (with *n*_*k *_= 100 or 200 mice) reared under a different environmental condition. This design allows QTL by environment interaction tests. For each mouse from each study group, the number of cancer cells is simulated at eight successive ages (*T *= 8) by assuming a multivariate normal distribution with environment-specific mean vectors specified by the logistic equation (1) and environment-specific covariance matrices specified by the AR(1) model. The parameters that fit the logistic equations and AR(1)-structured matrices are given in Table 1. Although the marker-QTL genotype frequencies are identical for the two groups, the effects of the QTL may be different because of the impact of environment on gene expression. Thus, the two groups are assumed to have different curve parameters for the same QTL genotype (Table 1). The residual variance is determined on the basis of heritability. For each group, two levels of heritability, 0.1 and 0.4, are assumed for the number of cancer cells at a middle time point.

**Table 1:** Maximum likelihood estimates of the parameters describing the clonal expansion, each corresponding to a QTL, and marker allele frequency, QTL allele frequency and marker-QTL linkage disequilibrium with 8 time points. Numbers in parentheses are the sampling errors of the estimates

		*n *= 200		*n *= 400	
			
Para-meters	True value	*H*^2 ^= 0.1	*H*^2 ^= 0.4	*H*^2 ^= 0.1	*H*^2 ^= 0.4
*P*	0.5	0.48(0.023)	0.49(0.021)	0.49(0.014)	0.50(0.012)
*Q*	0.6	0.62(0.014)	0.61(0.011)	0.61(0.011)	0.60(0.010)
*D*	0.08	0.074(0.0067)	0.075(0.003)	0.079(0.004)	0.079(0.004)
					
$K_{2}^{\left(1\right)}$	100	102.50(0.452)	102.31(0.449)	101.65(0.450)	100.59(0.441)
$r_{2}^{\left(1\right)}$	0.1	0.097(0.00069)	0.097(0.00062)	0.098(0.00061)	0.099(0.00012)
$K_{2}^{\left(2\right)}$	110	108.56(0.492)	109.20(0.481)	109.98(0.486)	110.25(0.479)
$r_{2}^{\left(2\right)}$	0.15	0.147(0.0011)	0.147(0.0011)	0.149(0.0010)	0.150(0.0007)
					
$K_{1}^{\left(1\right)}$	150	152.89(0.865)	152.35(0.862)	151.72(0.862)	150.06(0.858)
$r_{1}^{\left(1\right)}$	0.2	0.209(0.0015)	0.21(0.0014)	0.21(0.0014)	0.20(0.0011)
$K_{1}^{\left(2\right)}$	160	163.09(0.106)	162.52(0.105)	161.08(0.102)	160.32(0.098)
$r_{1}^{\left(2\right)}$	0.25	0.244(0.0016)	0.244(0.0016)	0.248(0.0012)	0.250(0.0011)
					
$K_{0}^{\left(1\right)}$	200	198.26(0.756)	199.63(0.752)	199.90(0.743)	200.03(0.735)
$r_{0}^{\left(1\right)}$	0.25	0.247(0.0053)	0.248(0.0050)	0.248(0.0051)	0.249(0.0046)
$K_{0}^{\left(2\right)}$	210	209.56(0.685)	209.79(0.682)	209.98(0.681)	210.22(0.668)
$r_{0}^{\left(2\right)}$	0.30	0.311(0.002)	0.311(0.001)	0.308(0.001)	0.302(0.0008)
					
*ρ*_1_	0.60	0.61(0.0078)	0.61(0.0076)	0.61(0.0071)	0.61(0.0070)
*ρ*_2_	0.60	0.593(0.0022)	0.595(0.0021)	0.595(0.0021)	0.598(0.0020)
*σ*_1_	1.31	1.322(0.0052)		1.319(0.0045)	
	0.538		0.539(0.0026)		0.538(0.0019)
*σ*_2_	1.31	1.322(0.0072)		1.320(0.0056)	
	0.53		0.53(0.0018)		0.53(0.0011)

The simulated data were analyzed by the model, which was repeated 100 times to estimate the means and sample errors of the MLEs of parameters. The estimation results are tabulated in Table 1. It can be seen that the QTL controlling age-dependent clonal expansion can be detected using the marker in association with the QTL. As expected, the frequencies of marker alleles can be estimated more precisely than those of QTL alleles. The precision of estimating QTL allele frequencies and marker-QTL linkage disequilibrium increases with increasing sample size and increasing heritability (Table 1). The curve parameters that describe age-specific cancer incidence can be generally well estimated, with increasing precision when sample size and heritability increase. A similar trend was found for the AR(1) parameters that model the structure of the covariance matrices.

**Figure 1:**
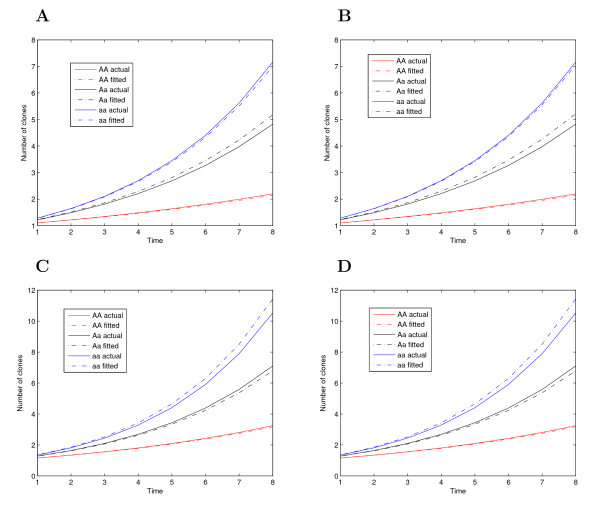
**Curves for the number of cancer clones changing with age, determined by three different QTL genotypes *AA*, *Aa*, and *aa*, using given parameter values (solid) and estimated values (broken) with different heritabilities (*H*^2^) for a sample size of *n *= 200.** (**A**) Group 1, *H*^2 ^= 0.1, (**B**) Group 1, *H*^2 ^= 0.4, (**C**) Group 2, *H*^2 ^= 0.1, and (**D**) Group 2, *H*^2 ^= 0.4.

**Figure 2:**
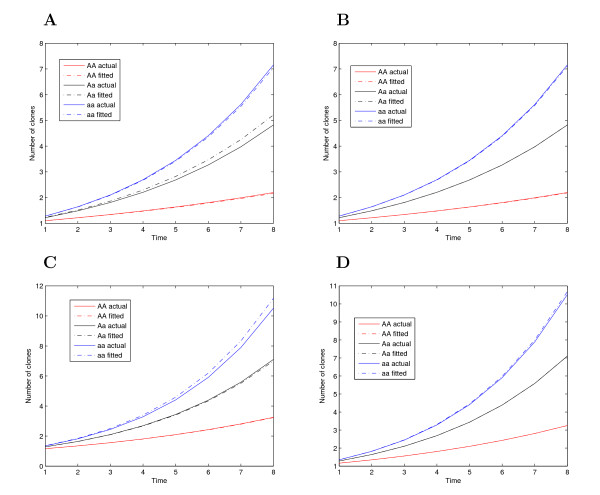
**Curves for the number of cancer clones changing with age, determined by three different QTL genotypes *AA*, *Aa*, and *aa*, using given parameter values (solid) and estimated values (broken) with different heritabilities (*H*^2^) for a sample size of *n *= 400.** (**A**) Group 1, *H*^2 ^= 0.1, (**B**) Group 1, *H*^2 ^= 0.4, (**C**) Group 2, *H*^2 ^= 0.1, and (**D**) Group 2, *H*^2 ^= 0.4.

Figures 1 and 2 illustrate the shapes of estimated age-dependent cancer incidence curves for each QTL genotype, comparing with those of given curves. In general, the estimated curves are consistent with those given curves even when the heritability (0.1) and sample size (200) are modest, suggesting that the model can reasonably detect the genetic control of cancer incidence curves. In practice, our model can formulate a number of meaningful hypotheses, e.g., (7)-(9). In this study, these hypothesis tests were not performed because no real data are presently available.

## Discussion

Aging is associated with a number of molecular, cellular, and physiological events that affect carcinogenesis and subsequent cancer growth [8]. In both humans and laboratory animals, the incidence of cancer is observed to increases with age [1,2,6]. A clear understanding of the genetic and developmental control of age-related cancer incidence is needed to design an optimal drug for cancer prevention based on an patient's genetic makeup. Although cellular and molecular explanations for this phenomenon are available [30,31], knowledge about its genetic causes is very limited. In this article, we derive a computational model for mapping quantitative trait loci (QTLs) that control an age-related rise in cancer incidence. The model was founded on the idea of functional mapping [16-21,23,32] by implementing a logistic equation for the age-related progression of cancer cells that is derived from molecular and cellular processes related to the pathway of cancer formation [4,33].

Our model for QTL mapping was constructed for mouse models for two reasons. First, it is possible to count cancer cells of an experimental mouse in lifetime, which is crucial for studying the association between cancer and cellular senescence. Second, environmental exposure for the mouse that leads to tumorigenesis can be controlled so that the effects of QTL by environment interactions on cancer incidence can be characterized. The model is built on the premise of linkage disequilibrium (i.e., non-random association between different loci) that has proven useful for fine-scale mapping of QTLs [34]. A recent survey about linkage disequilibria with a natural population of mice in Arizona suggests that this population is suitable for fine-scale QTL mapping and association studies [26]. In humans, it is not possible to count cancer cells in a person's lifetime. However, the idea of our model can be modified for human cancer studies by sampling people with different ages ranging from young (e.g., 10 years) to old (e.g., 75 years). For each subject in such a sampling design, the number of cells in the clone due to accumulated mutations is counted at several subsequent ages (at least three years). Thus, we will have an incomplete data set in which cell numbers for all subjects are missing at some particular ages. Hou et al.'s [35] functional mapping model, which takes into account unevenly spaced time intervals and missing data, can be used to manipulate such an incomplete data set.

We model the effects of environment including those related to lifestyle exposures on age-specific increases in cancer incidence. When the sexes are viewed as different environments, it will be interesting to incorporate sex-specific differences in haplotype frequencies, allele frequencies and linkage disequilibrium [36]. Also, as a general framework, we model the association between one marker and one QTL, which is far from the reality in which multiple QTLs interact with each other in a complicated network to affect a phenotype [24]. However, our model can be easily extended to consider these possible genetic interactions and fully characterize the detailed genetic architecture of cancer incidence. Bayesian approaches that have been shown to be powerful for solving high-dimensional parameter estimation [37] will be useful for implementing genetic interactions between different QTLs into our model for mapping age-related acceleration of cancer incidence.

With the availability of high-density SNP-based maps in humans and experimental crosses of mice, QTL mapping has developed to a point at which genetic variants for complex traits can be specified at the DNA sequence level. Wu and colleagues developed a handful of computational models for associating the haplotypes constructed by a series of SNPs and complex traits [22,38-41]. By incorporating these haplotype-based mapping strategies into the model proposed here, we can characterize specific combinations of nucleotides that encode an age-related increase in cancer incidence. Although our model has not been used in a practical project because no real data are available for now, specific experimental designs can be launched to establish and test new hypotheses about cancer progression. All in all, our model should stimulate new empirical tests and help to perform cutting-edge studies of carcinogenesis by integrating the epidemiological pattern of cancer incidence, molecular processes that derive cancer formation and development, mathematical modeling of cellular dynamics and statistical analyses of DNA sequences.

## Authors' contributions

KD derived the equation, programmed the algorithm and performed computer simulations. RW conceived the idea and wrote the manuscript.
